# Subcellular detection of PEBCA particles in macrophages: combining darkfield microscopy, confocal Raman microscopy, and ToF–SIMS analysis

**DOI:** 10.1007/s13346-022-01128-3

**Published:** 2022-02-19

**Authors:** Antje Vennemann, Daniel Breitenstein, Elke Tallarek, Ýrr Mørch, Ruth Schmid, Martin Wiemann

**Affiliations:** 1 IBE R&D Institute for Lung Health gGmbH, Mendelstr. 11, 48149 Münster, Germany; 2 Tascon GmbH, Münster, Germany; 3 Department of Biotechnology and Nanomedicine, SINTEF Industry, Trondheim, Norway

**Keywords:** Bioimaging, Label-free detection, NR8383 cell, Alveolar macrophage, PEBCA

## Abstract

The detection of biomedical organic nanocarriers in cells and tissues is still an experimental challenge. Here we developed an imaging strategy for the label-free detection of poly (ethylbutyl cyanoacrylate) (PEBCA) particles. Experiments were carried out with phagocytic NR8383 macrophages exposed to non-toxic and non-activating concentrations of fluorescent (PEBCA NR668 and PEBCA NR668/IR), non-fluorescent (PEBCA), and cabazitaxel-loaded PEBCA particles (PEBCA CBZ). Exposure to PEBCA NR668 revealed an inhomogeneous particle uptake similar to what was obtained with the free modified Nile Red dye (NR668). In order to successfully identify the PEBCA-loaded cells under label-free conditions, we developed an imaging strategy based on enhanced darkfield microscopy (DFM), followed by confocal Raman microscopy (CRM) and time-of-flight secondary ion mass spectrometry (ToF–SIMS). Nitrile groups of the PEBCA matrix and PEBCA ions were used as suitable analytes for CRM and ToF–SIMS, respectively. Masses found with ToF–SIMS were further confirmed by Orbitrap-SIMS. The combined approach allowed to image small (< 1 µm) PEBCA-containing phagolysosomes, which were identified as PEBCA-containing compartments in NR8383 cells by electron microscopy. The combination of DFM, CRM, and ToF–SIMS is a promising strategy for the label-free detection of PEBCA particles.

**Graphical Abstract:**

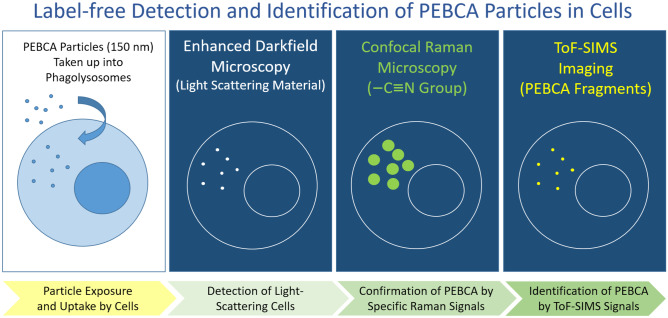

## Introduction

Nanoparticles are increasingly used for biomedical applications such as improved drug targeting [1]. Besides liposomes, polymeric nanoparticles are highly advantageous because they may act as stable carriers, e.g., for anti-cancer drugs (see for overview: [2]), therapeutic oligonucleotids [3], or siRNA [4]. Moreover, they have different and adaptable release kinetics, show a differential cellular uptake, and can cross the blood–brain barrier [5]. One class of polymeric nanoparticles is fabricated of poly (alkyl cyanoacrylate) (PACA), a polymer originally developed as a surgical glue. Later on, this material was discovered to be a versatile carrier for hydrophobic drugs to be injected into the blood circulation due to its high loading capacity for drugs and its tunable biodegradability [5]. PACA materials differ with respect to their alkyl side chains which may be composed of, e.g., butyl (PBCA), ethylbutyl (PEBCA), or octyl residues (POCA). Although there is some disparity of these matrix materials with respect to biopersistence and cellular responses [6], all of them can encapsulate hydrophobic drugs which are released upon intracellular digestion. Typically, PACA particles suited for i.v. drug delivery are smaller than 200 nm in diameter and can be designed to carry a surface coat built from different types of polyethyleneglycols (PEGs). This pegylation not only prolongs the circulation time of PACA particles in blood [7] but also governs, at least in part, the uptake into cells [8].

Understanding of the mechanisms of biodistribution is crucial to understand and further improve the targeting properties of PACA particles in the body. Rhodamine dyes have been used for studying the biodistribution of organic nanocarriers, but photobleaching and some loss of dye have been recognized as drawbacks [9]. For PEBCA particles, a fairly stable labelling with a hydrophobically modified Nile Red (NR668) was developed and verified in PC3 cells in vitro [10]. Fluorescent NR668-labelled PEBCA particles (PEBCA NR668) allowed to visualize the particle distribution also in vivo, at least at the organ level, and the i.v. administration of dye-labelled particles in mice led to an accumulation of fluorescence mainly in liver, spleen, lymph nodes, lung, and also in a patient-derived breast cancer xenograft [11]. However, it is also known that adding only small amounts of a dye may greatly alter the particles’ properties and also their cellular uptake [10]. Therefore, the development of label-free detection methods for medicinal particles in tissues is highly needed.

At present, the cell types that take up or even accumulate PACA particles in the body are not precisely known. Although in all these tissue cells of the reticulo-endothelial system including resident macrophages or dendritic cells may be dominantly involved in the uptake of blood-born particles [12], fluorescent PACAs have not yet been used to investigate the distribution at the cellular level in vivo. However, in vitro studies with PEBCA NR668 showed that the uptake by cancer cell lines is an active process involving caveolae- or clathrin-mediated processes [13]. Studies with PC3 and other cell types have furthermore revealed a dose-dependent cytotoxicity of PACA particles under in vitro conditions and this seems to be caused by the matrix material, with PEBCA being less toxic than PBCA or POCA [14]. Nevertheless, also the uptake of PEBCA induced an integrated stress response and fostered autophagy [15].

The aim of this study was to establish a detection method for PEBCA particles at subcellular resolution. The methods should be able to detect the matrix material PEBCA itself, such that addition of a fluorophore was no longer needed. Starting with cell culture experiments, the key elements of the method should be transferable to organ tissue sections.

Considering that tissue macrophages might be involved in the uptake of PEBCA particles which may also enter into the lung, we employed an alveolar macrophage model which has been established to investigate the bioactivity of (nano)particles and particles up to respirable sizes. The model comprises four assays describing cytotoxicity, activation, pro-inflammatory effects, and H_2_O_2_ generation and has been validated by inhalation studies with a set of 19 nanomaterials such as organic pigments, metal oxides, and carbonaceous materials [16, 17]. In a first step, we explored the biological activity of various PEBCA particles including dye- and cabazitaxel (CBZ)-loaded variants to define non-toxic particle concentrations for optimal particle exposure conditions. Then, the subcellular distribution of PEBCA particles was evaluated with a set of methods to establish the detection and identification of PEBCA in cells under label-free conditions. Starting with fluorescent PEBCA particles, we employed combinations of enhanced darkfield microscopy (DFM), hyperspectral imaging (HSI), confocal Raman microscopy (CRM), time-of-flight secondary ion mass spectroscopy (Tof–SIMS), and Orbitrap-SIMS. Finally, the PEBCA-containing ultrastructural elements to be visualized with these techniques were examined with transmission electron microscopy (TEM).

The study shows that a combination of methods is advantageous to detect PEBCA particles label-free and with adjustable subcellular resolution.

## Materials and methods

### Materials

PEBCA nanoparticles were synthesized under aseptic conditions at SINTEF (Trondheim, Norway) by mini-emulsion polymerization. Prior to synthesis, all solutions were sterile filtered, and all equipment was autoclaved. An oil phase consisting of poly (ethylbutyl cyanoacrylate) (PEBCA) (Cuantum Medical Cosmetics) containing 2 wt% Miglyol 812 (Cremer) and 10 wt% vanillin was prepared. For drug-loaded particles, the oil phase was added 12 wt% CBZ (BioChemPartner) and only 2 wt% vanillin was used. For dye-loaded particles, the oil phase was added NR668 (modified Nile Red, custom synthesis at SINTEF [18]). In a further modification, 0.2 wt% IR-780-Oleyl (custom synthesis at CEA LETI) plus 0.2 wt% NR668 was added to the oil phase.

An aqueous phase consisting of 0.1 M HCl containing the two PEG stabilizers (Brij®L23 and Kolliphor®HS15, both Sigma-Aldrich, 5 wt% of each) was added to the oil phase, immediately mixed and sonicated for 3 min on ice (6 × 30 s intervals, 60% amplitude, Branson Ultrasonics digital sonifier). The solution was rotated (15 rpm) at room temperature overnight. The pH was then adjusted to 5.0 to allow further polymerization for 5 h at room temperature. The dispersions were dialyzed (Spectra/Por dialysis membrane MWCO 100.000 Da) against 1 mM HCl to remove unreacted PEG. Particle size (z-average), polydispersity index (PDI), and zeta potential of the nanoparticles in phosphate buffer (10 mM, pH 7.0) were measured by dynamic light scattering (DLS) and laser Doppler micro-electrophoresis using a Zetasizer Nano ZS (Malvern Instruments).

To calculate the amount of encapsulated drug, the drug was extracted from the particles by dissolving them in acetone (1:10) and quantified by liquid chromatography coupled to mass spectrometry (LC–MS/MS) using an Agilent 1290 HPLC system coupled to an Agilent 6490 triple quadrupole mass spectrometer.

From these syntheses, we obtained the following stock solutions [concentration in brackets]: empty PEBCA particles (PEBCA, 79 mg/mL), PEBCA particles containing CBZ (PEBCA CBZ, 107 mg/mL), PEBCA particles containing NR668 (PEBCA NR668, 88 mg/mL), and PEBCA particles containing NR668 plus IR-780-Oleyl (PEBCA NR668 IR, 98 mg/mL). The latter particles had been designed for an animal study and were included in some early parts of the study and to test for a possible influence of the dyes on the Raman signal.

To study particle toxicity, we also included the lipidic particles Lipimage 815 for comparison (stock solution 100 mg/mL containing 0.26 wt% IR-780-Oleyl) which were received from CEA (Grenoble, France) in the course of the REFINE Project; due to the absence of specific Raman signals, we did not pursue on imaging experiments with these particles.

### Size determination of particles

**Fig. 1:**
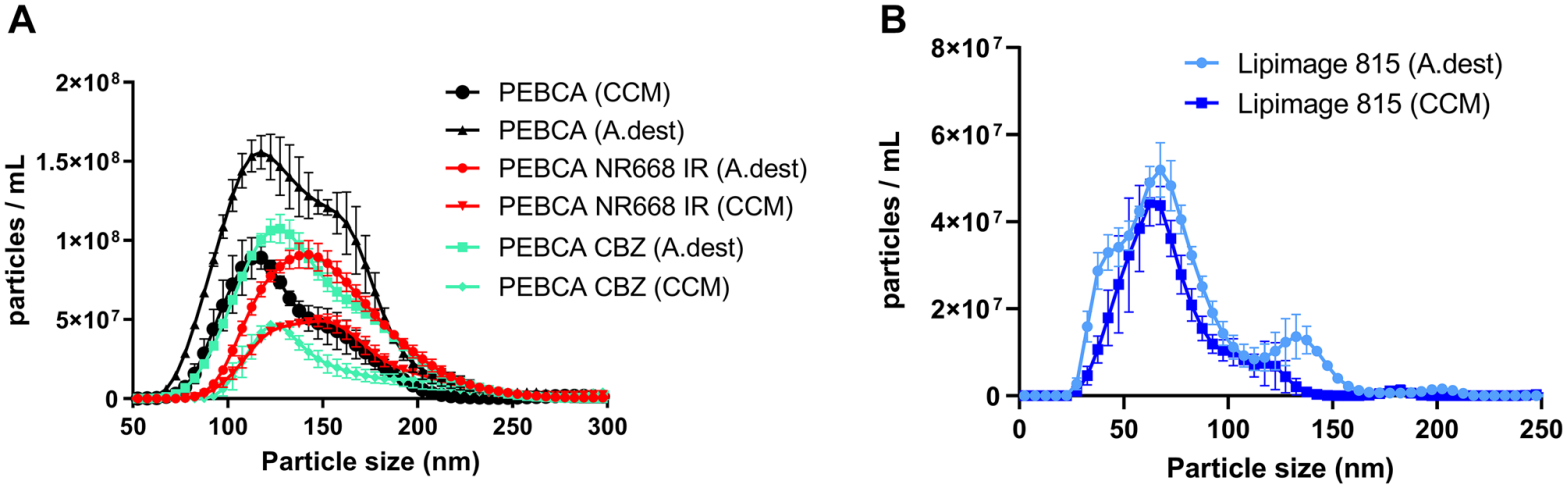
Hydrodynamic diameter of different PEBCA and Lipimage 815 particles. (**A**) PEBCA particles. (**B**) Lipimage 815 particles. Particles were dispersed in H_2_O (A. dest) or F-12 K cell culture medium (CCM) and analyzed with particle tracking analysis

The hydrodynamic diameter of Lipimage 815 and PEBCA particles was measured with particle tracking analysis (PTA) using a NanoSight LM10 instrument equipped with a blue laser (405 nm), an Andor CCD camera, and NanoSight software (NTA 3.1, Malvern Instruments GmbH, Herrenberg, Germany). Particles were diluted to measurable concentrations of approximately 5 × 10^8^ particles/mL using ultrapure H_2_O and F-12 K cell culture medium as a diluent. The hydrodynamic size of particles was 73.9 nm (Lipimage 815) and 138.4–164.6 nm (PEBCA variants) in H_2_O and F-12 K medium (Fig. 1).

### Cell culture experiments

NR8383 cells were maintained at 37 °C and 5% CO_2_ in 175 cm^2^ culture flasks in F-12 K medium (Sigma-Aldrich, Germany), supplemented with 15% heat-inactivated fetal calf serum, glutamine (2 mM), and 100 U penicillin and 10 mg/mL streptomycin. Half of the medium was replaced twice per week. For cell experiments, the aqueous nanoparticle stock suspensions were diluted either in F-12 K cell culture medium or Krebs–Ringer buffer (KRPG) supplemented with 2 mmol/L glucose. NR8383 cells were seeded in 96-well plates (3 × 10^5^ cells per well) and incubated with increasing concentrations of particle suspensions prepared in serum-free F-12 K medium for 16 h. F-12 K medium supernatants were retrieved and assayed for lactate dehydrogenase activity (LDH), glucuronidase activity (GLU), and tumor necrosis factor α (TNFα) as described [19]. LDH and GLU activities were normalized to the positive control value (100% value) obtained by adding 0.1% triton X-100 to an equal number of vehicle-treated cells. H_2_O_2_ concentrations were measured 90 min after adding the KRPG-diluted particle suspensions. All assays were carried out in triplicates and repeated three times. Vehicle-treated cells were used as negative controls. Cell-free wells were processed in the same way under cell culture conditions and used for background correction in colorimetric assays.

### Microscopy methods and confocal Raman microscopy

To identify and localize PEBCA particles in macrophages, NR8383 cells were detached from 96-well plates, suspended by pipetting, and centrifuged onto glass slides using a cytocentrifuge (Shandon Cytospin 3) run at 600 rpm for 6 min. Air-dried cells were then fixed with 4% phosphate-buffered formaldehyde for 10 min, rinsed twice in phosphate-buffered saline (PBS) and then in H_2_O. DFM plus fluorescence images were taken with an Olympus BX50 microscope equipped with an enhanced darkfield condenser, a 40 × objective, and a conventional fluorescence filter set for Texas Red fluorescence (Excitation: 535 nm, Emission > 620 nm). DFM plus HSI plus Raman images were taken with an Olympus BX43 microscope equipped with an enhanced darkfield condenser, a hyperspectral imaging device, and ENVI 4.8 software (CytoViva Inc., Auburn, AL, USA), and an integrated confocal Raman unit operated with LabSpec 6 software (Horiba Xplora Plus, HORIBA Jobin Yvon GmbH, Bensheim Germany). Cytospin preparations were viewed with a 60 × water immersion objective (Olympus, N.A. 0.9). Water immersion was mandatory as it allows DFM and inhibits the destruction of the sample by laser illumination during Raman imaging. A 532 nm Laser (120 mW) was operated with 100% laser power. Hyperspectral images were analyzed with a CytoViva plugin to ENVI 4.0 Software licensed to and distributed by CytoViva Inc. (CytoViva Inc., Auburn, AL, USA). Raman spectra were analyzed with LabSpec 6 software. Micrographs were taken either with a CCD camera (Retiga 4000R Fast 1394) operated with Q-Capture Pro software (Q-Imaging, Surrey, British Columbia, Canada).

### Time-of-flight secondary ion mass spectrometry

The ToF–SIMS measurements were performed using a TOF–SIMS.5 instrument (IONTOF GmbH, Münster, Germany) equipped with a 30 keV Bi-cluster liquid metal primary ion gun (LMIG) and a 20 keV gas cluster ion beam (GCIB). The latter was used to apply a mild sputter erosion of the sample prior to analysis by applying Ar_2000_^+^ clusters at 5 kV with an ion dose density of 1e^15^/cm^2^ in a raster field of 200 × 200µm^2^. For charge compensation, a flood gun was used. In addition, a sample flooding with Ar (1.6e^−6^ mbar) was applied. All imaging analyses were carried out using Bi_3_^+^ primary ions at a cycle time of 100 µs. The field of view was set to approximately 100 × 100 µm^2^ and the analysis area was scanned in random mode at least 60 times with a pixel number of 256 × 256. Analyses were performed in delayed extraction mode (extraction delay 0.045 µs) resulting in a mass resolution of > 5000 at m/z 86. Data evaluation was performed using Surface.Lab.7.2 (IONTOF GmbH, Münster, Germany).

### Orbitrap secondary ion mass spectrometry (Orbitrap-SIMS)

For Orbitrap-SIMS measurements, a M6 Hybrid-SIMS instrument was used (IONTOF GmbH, Muenster, Germany). Ar_2500_^+^ ions at 20 keV were used as primary ion species. The primary ion current was set to 83 pA and the primary ion dose density was limited to 1.2 e^14^/cm^2^. The He pressure in the collision cell was set to 6.7 e^−7^ mbar. RF values for the octupole were set to the following values: Oct 1/2: −30 V; Oct 3: −14 V; Oct 4: −9 V; Oct 5: −7 V. Nominal mass resolution of the Orbitrap was set to *R* = 240.000. An ion injection time of 2.9e^3^ ms was used. The adaptive ion injection system was disabled whereas the dataset denormalization was enabled. For analyses, 53 scans were acquired on a field of view of 300 × 300 µm^2^ with a defocused ion beam using a flood gun for charge compensation. Data evaluation was performed using Surface.Lab.7.2 (IONTOF GmbH, Münster, Germany).

### Transmission electron microscopy

Sterilized discs (diameter 6 mm) were punched from transparent Melinex film (Plano, Wetzlar, Germany), treated with 70% ethanol for 30 min, dried, and placed into the wells of a microtiter plate. Cells were seeded (3 × 10^5^ cells/well) in F-12 K medium with 5% serum and cultured on the discs. After 24 h, the medium was replaced by serum-free F-12 K medium containing 100 µg/mL of either PEBCA or PEBCA CBZ. Particle uptake was then continued for 4 and 16 h under cell culture conditions (37 °C, 5% CO_2_). Then, the cells were fixed with 2.5% glutardialdehyde in 0.1 M sodium phosphate buffer for 60 min. Cells were washed in PBS, post-fixed in 1% OsO_4_, stained en bloc with uranium acetate (1%), and embedded in Epon 812 (Sigma-Aldrich, Taufkirchen, Germany) as described earlier [20]. Isolated PEBCA particles were embedded in warm agar dissolved in phosphate-buffered saline (PBS) containing 3% bovine serum albumin and cooled on ice. Small pieces of the hardened mixture were embedded in Epon 812 and processed as described for the cells. Thin sections (50–60 nm) of all preparations were cut with a Reichert Ultracut microtome and viewed with a Tecnai G2 electron microscope at 120 kV. Digital images were taken with a Quemesa digital camera (Olympus Soft Imaging Solutions, Münster, Germany).

### Statistics

Results from LDH, GLU, TNFα, and H_2_O_2_ tests were compared to non-particle-treated controls by two-way analysis of variance (ANOVA) and Dunnets’s multiple comparisons test. Particle size data were compared using an unpaired *t*-test. Analyses were carried out with GraphPad Prism 6.01. For all experiments, *p* ≤ 0.05 was considered significant.

## Results and discussion

### Particle size measurement under cell culture conditions

In a first step, we measured the hydrodynamic diameter (HD) of all particles with PTA using H_2_O as a diluent and also in F-12 K medium to see whether there is an agglomeration under cell culture conditions. As shown in Fig. 1A, the HD of PEBCA, PEBCA NR668 IR, and PEBCA CBZ particles in F-12 K amounted to (mode values ± SEM) 114.8 ± 3.5 nm, 145 ± 4.7 nm, and 124.1 ± 0.6 nm, respectively. Lipimage 815 particles amounted to 64.7 ± 2.7 nm (Fig. 1B). Some particles showed a tendency to de-agglomerate upon transfer from H_2_O to cell culture medium and this effect is visible in Fig. 1 as a shoulder (PEBCA) or even a second smaller peak (Lipimage 815) which is present in H_2_O but not in F-12 K medium. Overall, all PEBCA and Lipimage 815 particles showed a good dispersability under cell culture conditions and retained their expected sizes.

### In vitro effects of particles on NR8383 alveolar macrophages

**Fig. 2:**
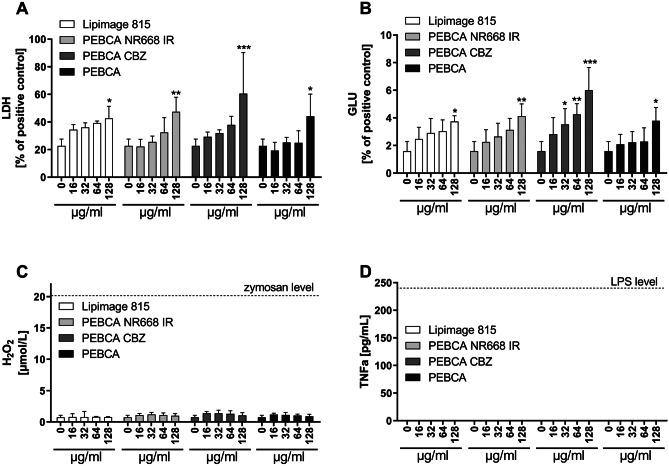
Cytotoxicity testing of PEBCA particles with NR8383 alveolar macrophages. Release of (**A**) lactate dehydrogenase (LDH), (**B**) glucuronidase (GLU), (**C**) H_2_O_2_, and (**D**) tumor necrosis factor α (TNFα) and elicited by the indicated concentrations of Lipimage 815, PEBCA, PEBC NR668, and PEBCA NR668 IR

PEBCA particles were administered to NR8383 cells under serum-free conditions. In the range of 16 to 128 µg/mL, all particles elicited a moderate release of lactate dehydrogenase (LDH) and glucuronidase (GLU) indicating membrane and lysosomal damage. Effects became significant upon ≥ 128 µg/mL (Fig. 2). There was no induction of TNFα or H_2_O_2_. Lipimage 815 particles showed a similarly low toxicity but somewhat flatter dose–response curves for LDH and GLU. Overall, these findings are in line with the effects of other organic particles of low toxicity such as pigments [17]. Considering all types of PEBCA particles, effects of PEBCA CBZ appeared somewhat more pronounced. CBZ is a cytostatic drug known to inhibit the depolymerization of microtubules (see also TEM study below) thus arresting the cell cycle. However, because NR8383 cells have a high serum demand and stop multiplying under serum-free conditions, the growth retarding effect of CBZ did not become evident here. Due to the beginning toxicity of PEBCA particles at 128 µg/mL, all imaging experiments were carried out with a concentration of 100 µg/mL which was regarded non-toxic during a 16-h incubation period.

### Imaging of particle uptake

**Fig. 3:**
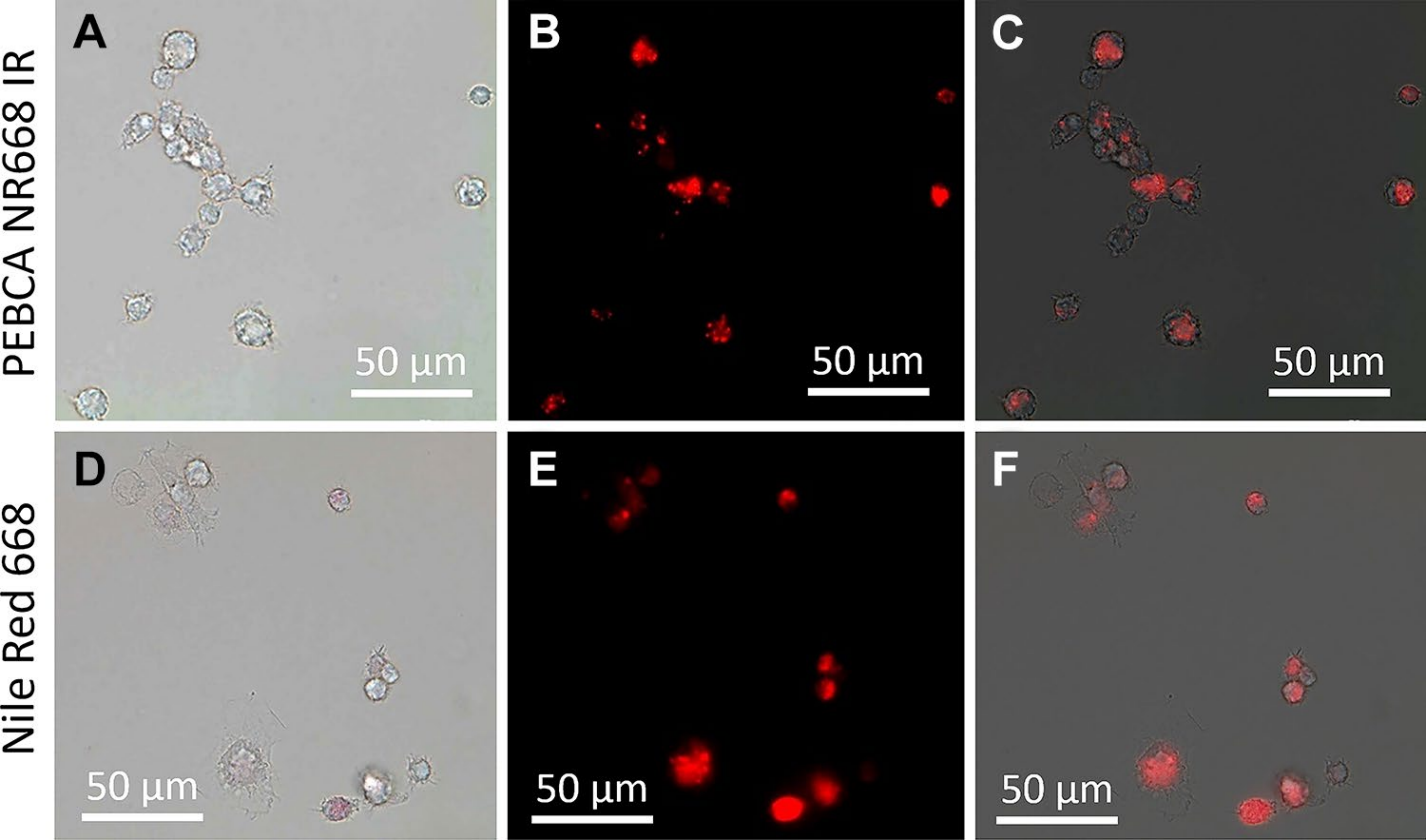
Uptake of PEBCA NR668 IR and hydrophobically modified Nile Red 668 by NR8383 cells. (**A**–**C**) Cells with dye-loaded PEBCA after 16 h. (**A**) Bright field image, (**B**) fluorescence image, (**C**) overlay from (**A**) and (**B**). (**D**–**F**) Cells stained with hydrophobically modified Nile Red 668 (5 µg/mL) after 16 h. (**D**) Bright field image, (**E**) fluorescence image, (**F**) overlay from (**D**) and (**E**)

Since PEBCA particles are transparent, fully dispersible, and not prone to agglomeration and/or gravitational settling, neither the particles themselves nor the particle load inside macrophages is visible by light or phase contrast microscopy under cell culture conditions. To verify particle uptake by NR8383 macrophages, we first administered fluorescent PEBCA NR668 IR. This led to a dotted labelling pattern in most though not all cells (Fig. 3). Surprisingly, also the dye NR668 dissolved in F-12 K medium led to a similar staining pattern, although in this case the fluorescence was more evenly distributed in the cytoplasm (Fig. 3B, E). Previous studies on cancer cell lines have shown that PEBCA NR668 are quite stable and retain their NR668 fluorescent label [10]. This, however, may be different in phagocytic cells. In any case, the staining results obtained with the isolated dye underlined the necessity to establish label-free methods to determine the cellular or even subcellular distribution of PEBCA particles in cells and tissues.

### Enhanced darkfield microscopy and hyperspectral imaging

**Fig. 4:**
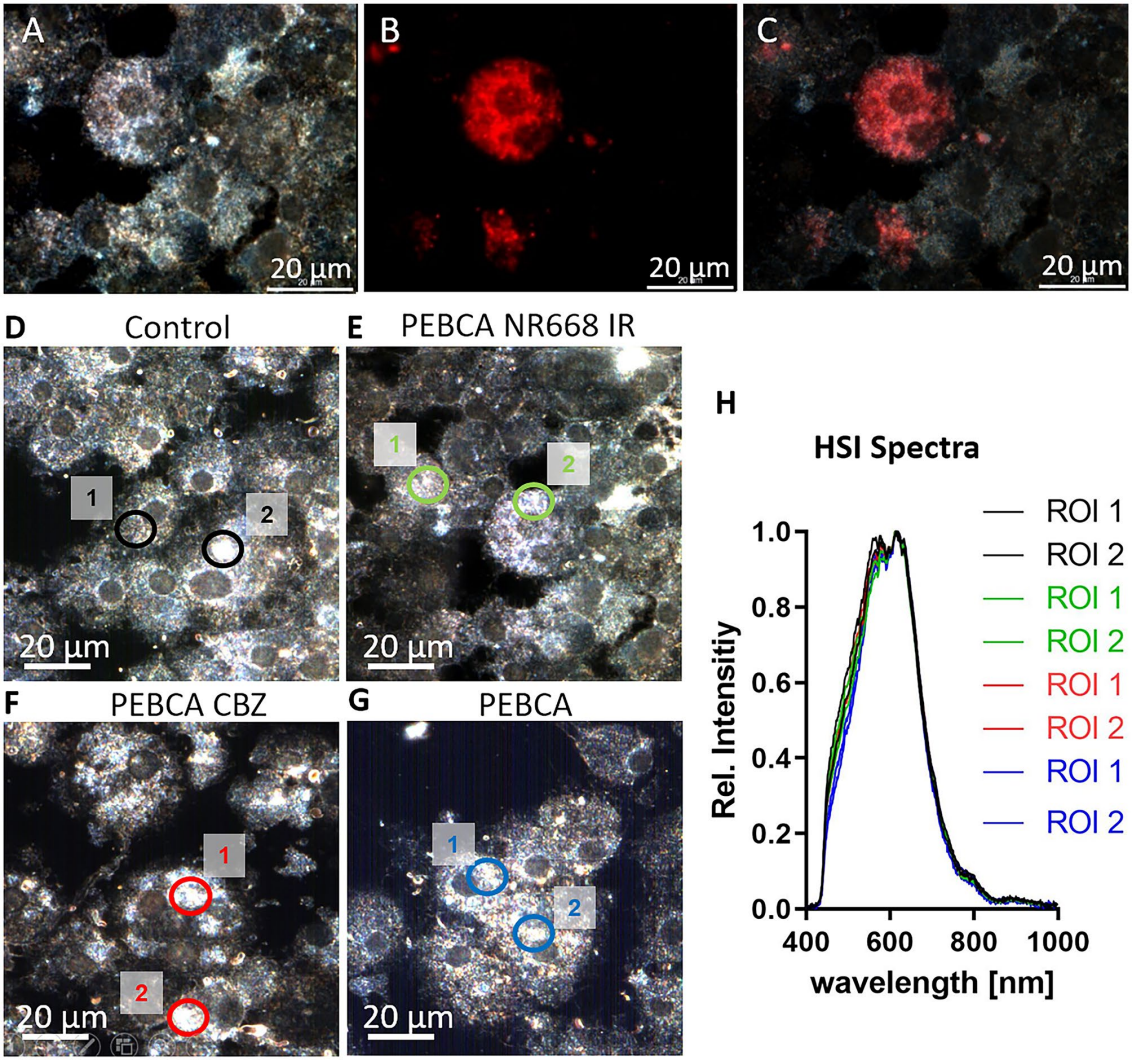
Enhanced darkfield microscopy and hyperspectral microscopy of NR8383 alveolar macrophages loaded with different PEBCA particles. Cells were loaded with 100 µg/mL of the indicated particles for 4 h. (**A**–**C**) Cells loaded with PEBCA NR668 IR. (**A**) DFM image, (**B**) fluorescence image, (**C**) overlay of (**A**) and (**B**). Note that light-scattering regions not always correspond to fluorescent areas. (**D**–**G**) Analysis of cells with DFM and hyperspectral imaging (HSI); two regions of interest (ROI) were analyzed in each picture. (**D**) Untreated control cells, (**E**) cells loaded with PEBCA NR668 IR, (**F**) cells loaded with PEBCA CBZ, (**G**) cells loaded with PEBCA. HSI curves from all regions 1 and 2 were superimposed. Despite some spectral disparity between 450 and 550 nm, we were unable to differentiate between differently treated cells (**H**)

Light-scattering (nano)objects inside cells can be viewed with DFM. This technique allows to detect, e.g., membrane-enclosed vesicles, border structures of nuclei, or lipid droplets but is more often used to detect metal-based nanoparticles such as silver or gold nanoparticles down to a size of 10–20 nm [21–23]. Figure 4 shows the DFM image of a NR8383 macrophage containing PEBCA NR668 IR side-by-side to the fluorescent image (Fig. 4A–C). The overlay image confirms that strongly light-scattering objects in the cytoplasm are largely congruent with the fluorescent label. Since this effect was found for CBZ-loaded as well as empty PEBCA particles, light scattering was most likely caused by the PEBCA matrix itself. However, there were also cells exhibiting increased light-scattering regions without any fluorescence, indicating that other structures than PEBCA particles may cause a similar degree of light scattering as well. Nevertheless, DFM was maintained as a useful screening tool to identify PEBCA-loaded cells with other methods.

In another attempt to differentiate PEBCA-loaded and control cells, we analyzed strongly light-scattering regions from untreated, PEBCA NR668 IR, and PEBCA CBZ-treated cells with hyperspectral imaging (HSI). This technique analyzes the hyperspectrum of each pixel of a microscopic image area. Unknown light-scattering objects may be identified with the help of spectral libraries of reference materials and analysis methods such as spectral angle mapping (SAM). Since the spectral libraries from the pure PEBCA materials revealed no differences (data not shown), we compared side by side the spectral libraries from control and PEBCA-loaded cells. This is a well-accepted method to circumvent artificial differences caused, e.g., by different illumination settings and/or chemical surroundings [24–26]. Unexpectedly, the hyperspectra were very similar and did not allow to distinguish between the four cell groups (Fig. 4D–H), such that HSI was no longer pursued as a method to identify PEBCA particles in cells.

### Confocal Raman microscopy

**Fig. 5:**
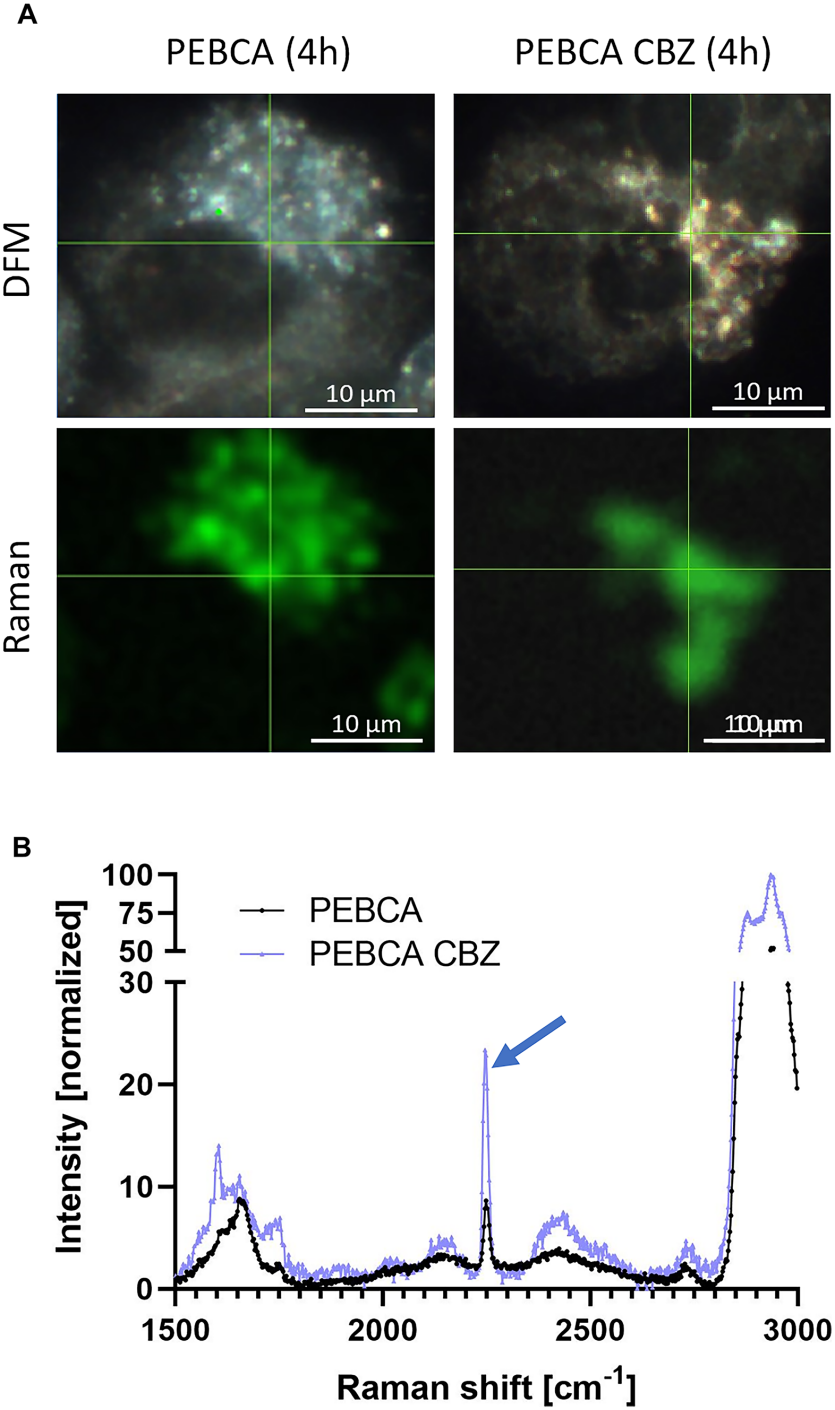
Confocal Raman microscopy of NR8383 macrophages loaded with PEBCA or PEBCA CBZ. Cells were exposed to indicated particles for 4 h, air-dried, and fixed with formalin. (**A**) Enhanced darkfield microscopy (DFM, upper panels) and CRM images of the same cells imaged for the Raman band of the nitrile group (2243 cm^−1^). Note that the numerous light-scattering cytoplasmic vesicles seen with DFM are co-localized with the Raman signal (green) below. (**B**) Raman spectra of the cells treated with PEBCA and PEBCA CBZ as shown in (**A**); the positions where spectra were taken are indicated by the cross lines in (**A**). Arrow points to the larger signal of the PEBCA CBZ-treated cell

Raman microscopy is a label-free imaging technique that is useful for the analysis of cells and tissues [27]. For the detection of biomedical nanocarriers in cells, so-called vibrational tags such as alkynes may be used, because their Raman signals fall into the so-called cell-silent region with wavenumbers ranging from 1800 to 2800 cm^−1^ [28, 29]. We found that the PEBCA matrix is similarly suited for confocal Raman microscopy as it contains nitrile groups whose Raman signal appears at the wavenumber 2243 cm^−1^ [30, 31]. While experiments with Lipimage 815 in NR8383 cells failed to deliver any characteristic Raman bands (data not shown), the nitrile band (wavenumber 2243 cm^−1^) was found in PEBCA- or PEBCA CBZ–loaded macrophages (Fig. 5 A,B), but not in control macrophages. Raman studies with pure PEBCA or PEBCA CBZ particles confirmed the occurrence of the signal of the nitrile group, which is part of the ethylbutyl cyanoacrylate moieties of the PEBCA molecule, the major component of the PEBCA particles. Figure 5A also shows that the 2243 cm^−1^ band was confined to cytoplasmic regions and that it was localized in regions bearing numerous light-scattering vesicles. Interestingly, PACA CBZ led to larger and more intensely light-scattering vesicles. All of these structures contained the Raman signal of PEBCA which was more pronounced in PEBCA CBZ- than in PEBCA-treated cells. At present, the reason for larger vesicles and higher Raman intensities in PEBCA CBZ–treated cells are not known. Because CBZ induces some alterations of the cytoskeleton (see TEM study below), it is, however, tempting to speculate that there is an (indirect) impact of released CBZ on vesicle transport and/or the phagolysosomal route. Further detailed investigations are needed to unravel underlying processes. At least the comparison of DFM and Raman images suggests that the light-scattering objects visible with DFM are PEBCA-containing vesicles. However, due to the limited resolution of CRM (Fig. 5A) signals may also represent PEBCA components within the cytoplasm. In order to describe the subcellular distribution of PEBCA in more detail, we employed ToF–SIMS and TEM.

## ToF–SIMS and Orbitrap-SIMS

**Fig. 6:**
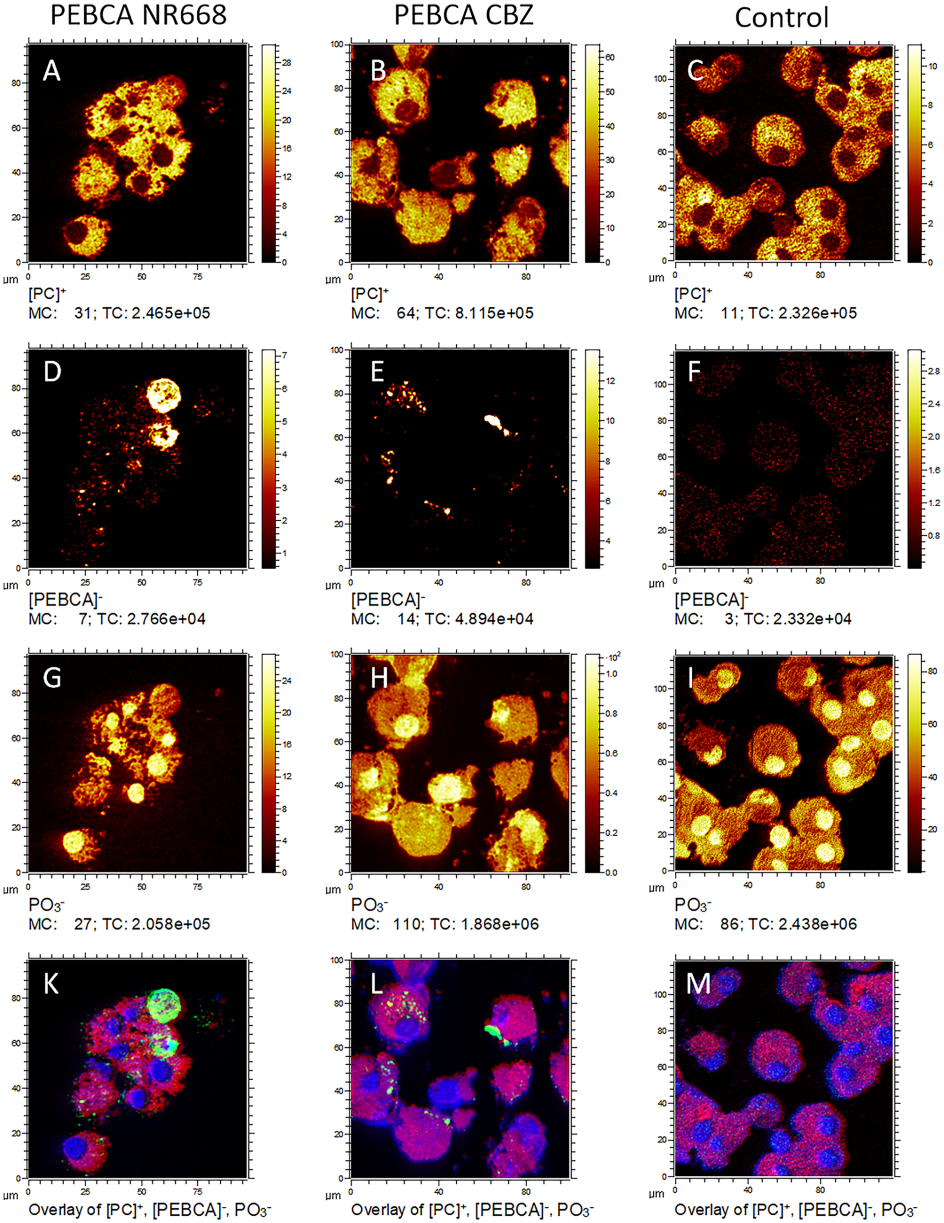
ToF–SIMS imaging of NR8383 cells treated with PEBCA particles. Air-dried and formalin-fixed cytospin preparations after 4 h of particle treatment. (**A**, **D**, **G**, **K**) PEBCA-NR668; (**B**, **E**, **H**, **L**) PEBCA CBZ; (**C**, **F**, **I**, **M**) untreated control cells. (**A**, **B**, **C**) Cytoplasmic signals of phosphatidylcholine from the sum of secondary cation intensities. (**D**, **E**, **F**) PEBCA signals from the sum of secondary anion ion intensities, and (**G**, **H**, **I**) nuclear signals from PO_3_^−^ only. (K, L, M) Red/green/blue correlation analysis of the respective signals (phosphatidylcholine/PEBCA/PO_3_^−^)

To achieve a molecular imaging of molecules identified by mass with high lateral resolution, we further analyzed PEBCA-loaded cells with ToF–SIMS. In a first step, cells were sputtered by a moderate dose of Ar-Clusters to remove the outer cell layers allowing to view inside the cells [32]. Cells were then analyzed by ToF–SIMS imaging in positive as well as in negative secondary ion polarity on the same position in delayed extraction mode [33]. Figure 6A–M shows the combined images from ToF–SIMS analyses of three different groups of secondary ion species:

1. Fragments of the phosphatidylcholine head group (C_3_H_8_N^+^, C_5_H_12_N^+^, C_5_H_15_NO_4_P^+^) [34] are found anywhere in the cell but are most intense in cytoplasmatic regions (Fig. 6A, B, and C). [32, 35].

2. Cell nuclei which are indicated by PO_3_^−^ (Fig. 6G, H, and I) [32, 35].
3. PEBCA-specific signals which represent the summed intensities of the three negatively charged secondary ions C_9_H_14_NO_2_^−^, C_13_H_17_N_2_O_2_^−^, C_19_H_27_N_2_O_4_^−^ are shown in Fig. 6D, E, and F. These ions most likely represent monomeric and dimeric fragments of the PEBCA polymer. High intensities of PEBCA signals were found in PEBCA NR668– and, even more pronounced, in PEBCA CBZ–treated NR8383 cells (which was in accord with DFM and Raman images, see above), but not in non-treated control cells (Fig. 6D–F). Typically, PEBCA signals were located in the cytoplasm outside the nuclei (Fig. 6K, L). However, we also found a few rounded cells within which the PEBCA signal was more or less evenly distributed throughout the cell (Fig. 6D). These cells are interpreted as dying cells because they were devoid of a (PO_3_^−^ positive) nucleus. Due to the overall high intensity of the PEBCA signal, it cannot be excluded that excessive particle load has contributed or even caused cell death in these cases. However, these events were rare and not reflected by the moderate release of LDH and GLU (see Fig. 2). We assume that the intense signal of PEBCA-loaded dying cells is caused by intact or degraded PEBCA which is no longer compartmentalized in phagolysosomes. In contrast, untreated control cultures hardly contained any dying cells and no concentrated PEBCA signals. Instead, we found, at a low level, background signals which were evenly scattered throughout the cells (Fig. 6F). These signals most likely result from mass interferences with cellular molecules as suggested by Orbitrap-SIMS data (see below).

**Fig. 7:**
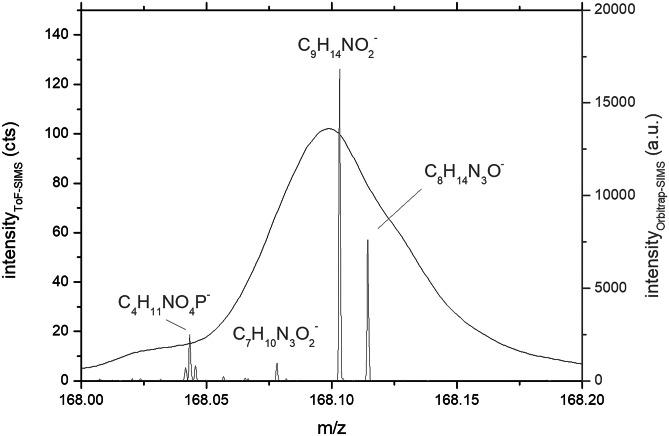
Comparison of ToF–SIMS and Orbitrap-SIMS spectra from a PEBCA NR668–loaded NR8383 cell. Note the highly increased resolution of the Orbitrap-SIMS spectrum (peak signals) compared to the Orbitrap-SIMS spectrum (curve). See text for further explanation

**Fig. 8:**
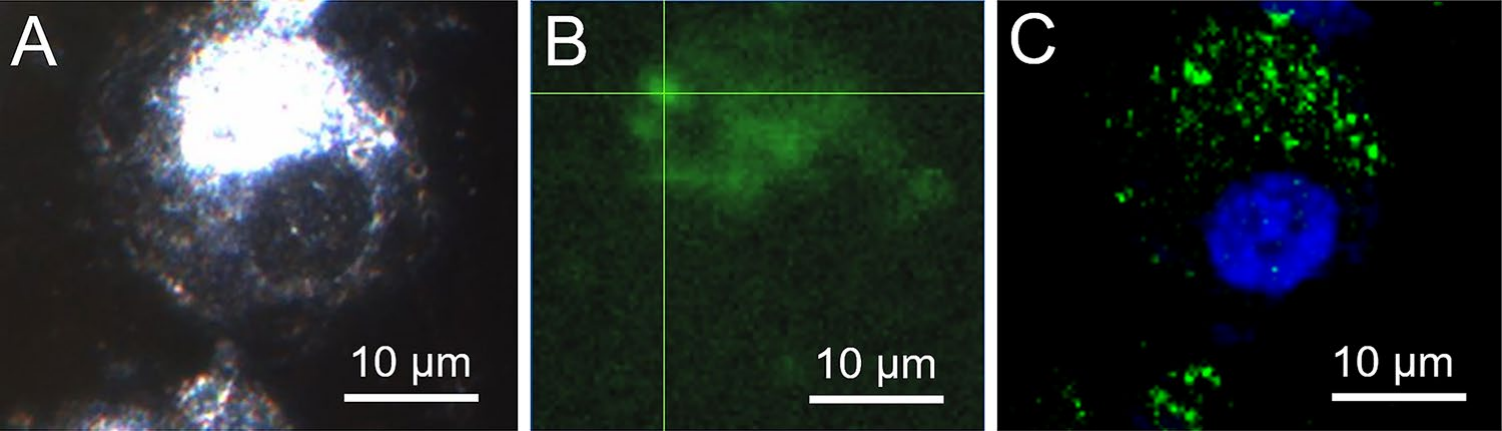
ToF–SIMS imaging of PEBCA CBZ–loaded NR8383 cells in comparison to DFM and CRM. Images in (**A**–**C**) were taken from the same cells. (**A**) DFM, (**B**) CRM, (**C**) Tof–SIMS of the PEBCA^−^ (green) and the nuclear PO_3_^−^ signal (blue); the PEBCA intensity scale was adapted to show small spots

In order to increase the reliability of the peak assignment to *m*/*z* values and to reveal mass interferences, Orbitrap-SIMS spectra of the same samples were measured. One example is shown in Fig. 7 for a PEBCA NR668–containing cell showing that the ToF–SIMS signal at *m/z* 168.1 is attributed to the PEBCA signal C_9_H_14_NO_2_^−^. Due to higher mass resolution (5000 vs. 168.000) and mass accuracy (0.9 ppm vs. 50 ppm) compared to the ToF–SIMS imaging, the Orbitrap-SIMS-spectrum revealed additional peaks whose underlying masses cannot be mass-separated in the ToF–SIMS imaging approach but, nevertheless, contributed to the non-zero background shown in Fig. 6F. At present, the complete mass separation of C_9_H_14_NO_2_^−^ from C_8_H_14_N_3_O^−^ and C_7_H_10_N_3_O_2_^−^ (all most likely PEBCA signals, see Figs. 7, 8) and C_4_H_11_NO_4_P^−^ (Fig. 7, most likely a phosphatidylcholine signal) is not possible in the ToF–SIMS imaging approach of PEBCA particles in cells and contributes to the lower detection limit of the method. With respect to the identification of PEBCA-contained molecules, it should be underlined that neither the fluorophores NR668 and IR-780-Oleyl nor CBZ-specific signals were detected in the ToF–SIMS approach. However, the molecular ion of NR668 was detected within the Orbitrap-SIMS spectrum in low intensities (data not shown).

We also compared Tof–SIMS images directly to those of DFM and CRM (Fig. 8). Therefore, fixed PEBCA CBZ–loaded macrophages were selected with DFM before a PEBCA burden was confirmed with CRM (both under water immersion). The sample was then dried, and identical positions were inspected with ToF–SIMS. Figure 8A–C shows that the highly light-scattering region of DFM corresponds to the region of the Raman signal representing the nitrile group. Unlike the Raman image, ToF–SIMS now provides full structural details showing the PEBCA-containing vesicles down to the submicron range.

**Fig. 9:**
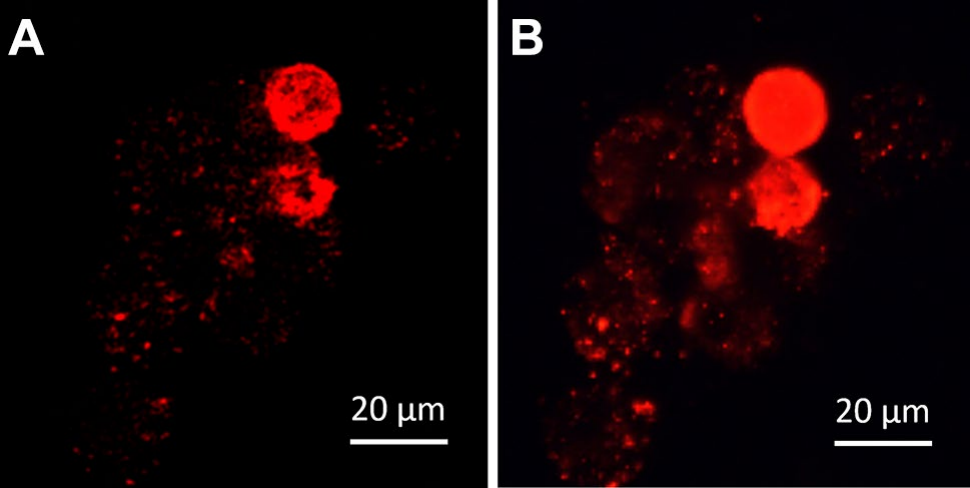
ToF–SIMS imaging of PEBCA NR668–loaded NR8383 cells in comparison to fluorescence microscopy. Both images were taken from the same cell group treated with PEBCA NR668. (**A**) PEBCA signal distribution (red); the intensity scale was adapted to show small spots. (**B**) Fluorescence image

Finally we compared ToF–SIMS imaging and fluorescence microscopy using PEBCA NR668–loaded cells. Figure 9 shows that ToF–SIMS imaging may reach a higher resolution than conventional fluorescence microscopy (FLM). However, when comparing both images, one needs to consider that the origin of the signal differs in both set-ups. In the FLM approach, the distribution of the fluorophore NR668 is projected onto the target of a CCD camera, while in ToF–SIMS, the distribution of the PEBCA polymer is probed. While autofluorescence and out-of-focus fluorescence reduce the contrast of the (non-confocal) fluorescent image, it is mass interference which reduces the image quality in ToF–SIMS. With respect to lateral resolution, the pixel size of the ToF–SIMS image is 380 nm^2^, which is close to what a confocal microscope can reach. Thus, although both approaches lead to highly similar images, it needs to be underlined that the ToF–SIMS approach is a label-free method, which contains chemical information. Moreover, it can be applied to all PEBCA particles irrespective of additional labels. Therefore, ToF–SIMS is a highly relevant technique for medical bioimaging and capable to show the biodistribution of unlabelled PEBCA particles.

## Transmission electron microscopy

**Fig. 10:**
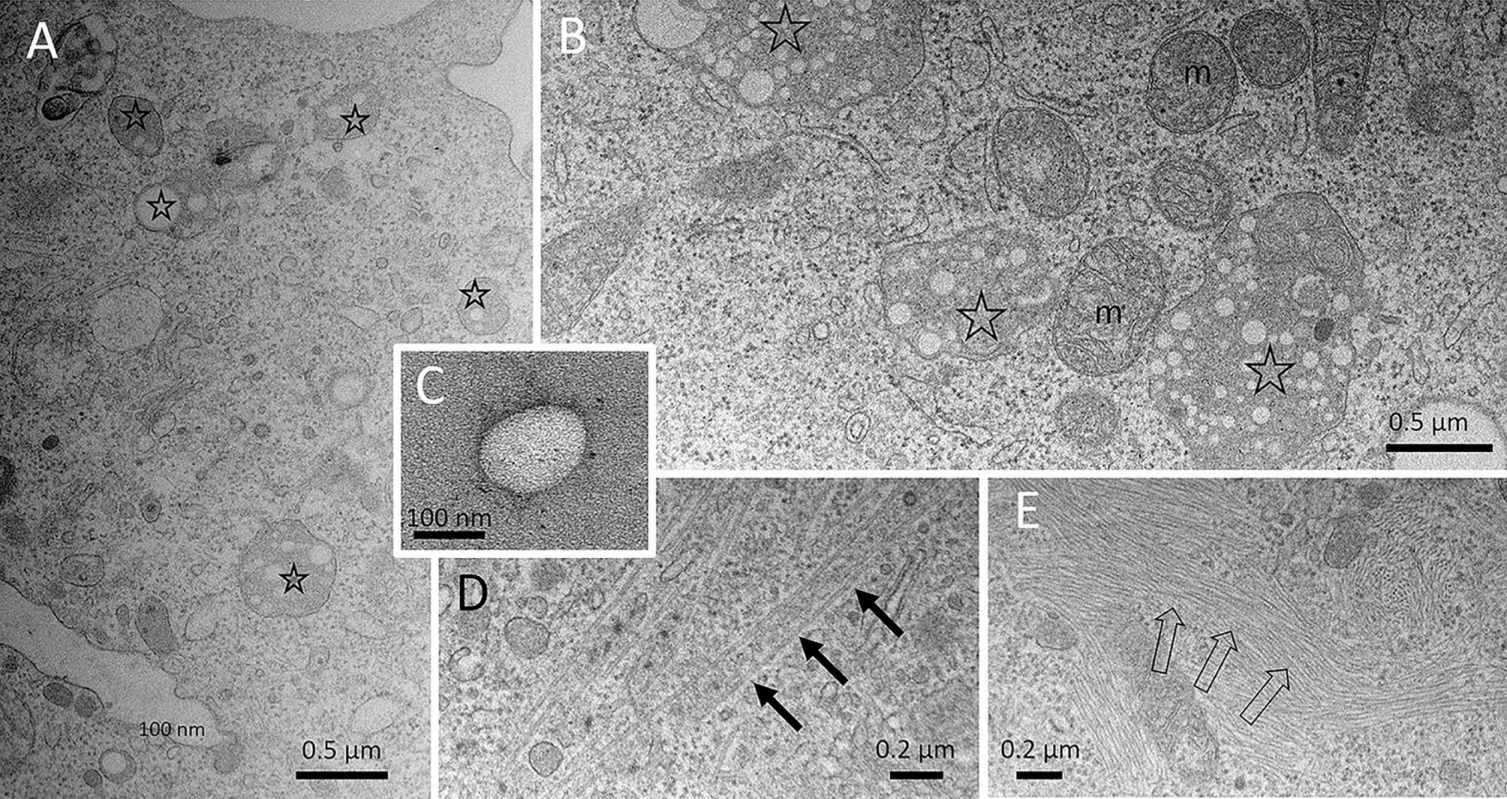
Transmission electron microscopy of PEBCA particles inside NR8383 macrophages. Cells were exposed to PEBCA (100 µg/mL) for 4 h (**A**) and 16 h (**B**); phagolysosomes filled with numerous electron-translucent spheres (open asterisks) become more prominent over time. m, mitochondrium. (**C**) Isolated PEBCA particle; its outer circumference carries electron dense structures. (**D**, **E**) PEBCA CBZ-treated cells often contained bundles of microtubules (**D**, arrows) or prominent microfilaments (**E**, open arrows)

Considering the accumulation of light-scattering vesicles and the dotted-labelling patterns found with FLM and ToF–SIMS, we were curious about the fine structural details in PEBCA-loaded cells and employed transmission electron microscopy (TEM) to PEBCA- and PEBCA CBZ–loaded cells. We found characteristic membrane-bound vacuoles within the cytoplasm of NR8383 macrophages which contained cross sections of translucent spheres (diameter mostly < 200 nm) embedded in a homogeneous electron dense matrix (Fig. 10A–C). The circumference at least of some of these spheres was more electron dense, as typically found for isolated PEBCA CBZ particles embedded in a BSA matrix (Fig. 10C), and possibly represents the PEG coat of the vesicles. As found with light microscopy and ToF–SIMS, the number and size of these vacuoles apparently increased from 4 to 16 h (Fig. 10A–B). Despite careful examination, we were unable to detect sphere-like objects freely in the cytoplasm or within any other cellular compartment such as the nucleus, suggesting that PEBCA particles do not pass membrane structures unless a cell undergoes, e.g., necrosis. Due to the size distribution of included spheres, the electron density of the surrounding matrix, and the absence of similar vacuoles in untreated controls and also in many NR8383 cells investigated in previous studies [20], the vacuoles of Fig. 10A, B were interpreted as PEBCA particle-filled phagolysosomes.

It is also noteworthy that PEBCA-loaded cells examined by TEM did not show any change or damage of the cellular ultrastructure. However, in some PEBCA CBZ–treated cells, we found increased amounts of microtubules (Fig. 10E), which is an untypical finding for NR8383 macrophages. Since CBZ inhibits the depolymerization of microtubules and may also contribute to programmed cell removal and NfkB activation in macrophages [36], assemblies of microtubules and intermediate filaments (Fig. 10E) are interpreted as CBZ effects.

Overall, the TEM findings confirm the strictly vesicular localization of PEBCA particles in intact cells and suggest a phagolysosomal degradation pathway.

## Conclusion

A detailed understanding of how PACA particles accumulate within cells of a targeted tissue is necessary to fully understand their biodistribution, to optimize the administered dose, to overcome side effects, and eventually to reach regulatory approval. To this end, we developed a label-free imaging strategy for PEBCA particles in cells. NR8383 macrophages were taken because they resemble other resident phagocytic cells in the liver, spleen, or lymph nodes and because they are capable to engulf and accumulate nanosized organic particles. Starting with fluorescent PEBCA NR668 particles, we noticed a differential loading of the cells imposing the challenge to identify cells with a promising particle load also under non-fluorescent conditions. DFM combined with CRM was found as a method of choice which allowed to identify PEBCA particles by the Raman spectrum of nitrile groups. Technically, this was achieved with water immersion objective mounted to a combined DFM-Raman microscope system. While this two-step approach was already sufficient to describe the subcellular distribution of PEBCA in cells, in depth analysis of promising regions of interest was possible on the same sites using ToF–SIMS and Quadrupol-SIMS. These techniques not only confirmed the chemical nature of PEBCA particles but furthermore revealed the subcellular distribution of the material at a high resolution.

With respect to the universality of CRM, it should be stressed that the method may be easily transferred to other functional groups (e.g., alkynes) whose Raman signals also lie in the so-called cell-silent region (1800–2800 cm^−1^) where cells and tissue have no inherent Raman signals as outlined before [30]. Concerning ToF–SIMS and Orbitrap-SIMS, many other molecules may be suited for the detection of a given polymer, provided that ions with characteristic mass are generated which can be imaged against the background of cellular constituents.

The method should also be easily transferable to cryo-sections of routinely fixed organs from PEBCA-treated animals, because cells had been fixed with a routinely used concentration of formaldehyde. This preserved PEBCA-filled vesicles in cells and allowed their imaging with subcellular resolution. Also, it did not compromise the Raman signals falling into the cell-silent region. Although we are unable to provide a value for the lower limit of detection (LOD), a high sensitivity is to be expected because small (ca. 1 µm) vesicles, whose structures were confirmed by TEM, were detectable with ToF–SIMS and, under ideal conditions, also with CRM. Since both techniques also allow for the 3D bioimaging of major cellular compartments (such as nuclei, cytoplasm), PACA particles can be detected with high precision at the subcellular level. Future instrumental developments are expected to even improve the resolution of both CRM and ToF–SIMS. Overall, the label-free imaging strategy developed in this work could substantially contribute to the new field of visual medicine.

## Data Availability

Data and materials may be obtained via the corresponding author upon request.
